# Moyamoya disease masquerading as CNS vasculitis: Diagnostic challenges and imaging insights

**DOI:** 10.5339/qmj.2026.68

**Published:** 2026-09-22

**Authors:** Areej M Saad, Ahmed Hatim Mohamed Ahmed, Hana Elamin, Abdelrahman Hamad, Gamal Alfitori, Vivian Zuzel Rodriguez Sanchez, Elhassan M Abdalla, Aiman E Aldhubaibi

**Affiliations:** 1 Department of Medicine, Hamad General Hospital, Hamad Medical Corporation, Doha, Qatar; 2 General Medicine, Hamad Medical Corporation, Doha, Qatar

**Keywords:** Moyamoya disease, central nervous system vasculitis, cerebrovascular disorder, imaging, vessel wall imaging, Qatar

## Abstract

**Background:**

Moyamoya disease (MMD) is a rare, progressive cerebrovascular disorder that can mimic central nervous system (CNS) vasculitis on imaging, particularly when advanced imaging techniques, such as vessel wall imaging, are used; thereby creating significant diagnostic uncertainty.

**Case Presentation:**

A 38-year-old woman presented with a one-week history of headache and binocular diplopia. Neuroimaging demonstrated a subacute right occipitoparietal infarct. Magnetic resonance angiography showed multifocal stenoses with extensive collateral vessels. High-resolution vessel wall imaging demonstrated circumferential enhancement, initially raising concern for CNS vasculitis; however, cerebrospinal fluid analysis was non-inflammatory, and autoimmune and infectious panels were negative. Digital subtraction angiography confirmed bilateral terminal internal carotid artery stenosis with lenticulostriate collaterals, consistent with MMD. The presence of a positive family history of MMD further supported the diagnosis. She improved clinically, was discharged on aspirin, and remained asymptomatic at neurosurgery follow-up.

**Discussion:**

This case highlights the diagnostic challenges in differentiating MMD from CNS vasculitis. The presence of circumferential vessel wall enhancement, which is not specific and may overlap with inflammatory vasculopathies, illustrates how reliance on vessel wall imaging alone can be misleading. This case emphasizes the importance of integrating clinical, laboratory, radiological, and familial data to distinguish non-inflammatory arteriopathies from true vasculitis accurately. Similar diagnostic dilemmas have been reported in the literature, emphasizing the need for careful evaluation to avoid unnecessary immunosuppressive treatment.

**Conclusion:**

Differentiating MMD from CNS vasculitis requires careful integration of clinical, radiological, and familial data to prevent misdiagnosis and unnecessary immunosuppression. A structured, multimodal diagnostic approach is essential, particularly when imaging findings are atypical or potentially misleading. Early recognition and appropriate management can lead to favorable outcomes, as illustrated by our patient, who showed complete recovery and remained symptom-free on medical therapy.

## 1. INTRODUCTION

Stroke in young adults is an increasingly recognized clinical problem, with a significant proportion attributed to intracranial large vessel stenosis and non-atherosclerotic cerebrovascular disorders.^1^ These conditions can present overlapping clinical and radiological features, often posing diagnostic challenges. Among these, Moyamoya disease (MMD) is a rare, progressive, non-inflammatory cerebrovascular arteriopathy characterized by progressive stenosis of the intracranial internal carotid arteries and their proximal branches, which may mimic central nervous system (CNS) vasculitis on imaging.^2,3^

Although classically reported in East Asia, with prevalence in Japan estimated at 3.2–10.5 per 100,000,^2^ MMD has been increasingly recognized worldwide, including in non-Asian populations.^3,4^ Clinical manifestations range from ischemic strokes and transient ischemic attacks (TIAs) to seizures or chronic headaches.^2,3^ Diagnosis is confirmed by digital subtraction angiography (DSA), while non-invasive modalities, such as magnetic resonance angiography (MRA) and computed tomography angiography (CTA), are often used in initial evaluation.^5^ Differentiating MMD from CNS vasculitis remains particularly challenging due to overlapping imaging features, such as multifocal arterial stenoses and the presence of collateral vessels. In recent years, high-resolution vessel wall imaging (HR-VWI) has been increasingly utilized to aid in this distinction; however, enhancement patterns may be variable and non-specific, potentially leading to diagnostic uncertainty.^6,7^

Accurate differentiation between MMD and CNS vasculitis is critical, as management strategies differ substantially. Misdiagnosis may result in inappropriate immunosuppressive therapy, whereas patients with MMD benefit from antiplatelet therapy and, in selected cases, surgical revascularization.^5,8^

While several reports have described the overlap between MMD and CNS vasculitis, diagnostic challenges persist, particularly when imaging findings are atypical or misleading. This case highlights a diagnostically challenging presentation in which circumferential vessel wall enhancement initially suggested an inflammatory vasculopathy, emphasizing the importance of integrating clinical, laboratory, radiological, and familial data to reach an accurate diagnosis.

## 2. CASE PRESENTATION

We present the case of a 38-year-old woman of Indian origin, with no known past medical history, who self-presented to the emergency department after a seven-day history of headache and visual disturbances. The headache was intermittent, tension-like in nature, and localized to the temporal region, partially relieved with analgesics. The visual complaint was binocular diplopia, described as overlapping of images. She was initially evaluated by the Ophthalmology team, who performed a fundoscopy and documented no optic disc edema. Due to persistent symptoms, she was referred back to the emergency department for further evaluation and admitted under the Neurology team.

A non-contrast computed tomography (CT) scan of the brain revealed a hypodense lesion in the right occipitoparietal lobe with loss of gray–white matter differentiation and mild mass effect, raising concern for ischemia versus a neoplastic lesion, with surrounding edema (Figure 1). The neurosurgery team found no indication for urgent surgical intervention and planned reassessment following magnetic resonance imaging (MRI), while the neurology team advised withholding aspirin and statin and proceeding with MRI for further evaluation.

(MRI of the brain, including perfusion-weighted imaging and magnetic resonance spectroscopy (Figures 2A–D), as well as post-contrast sequences (Figures 3A and 3B), demonstrated a subacute infarct in the right occipital cortical and subcortical regions extending slightly into the adjacent posterior temporal and parietal lobes. There was no evidence of hemorrhagic transformation, mass lesion, or hydrocephalus (Figures 4A and 4B). Magnetic resonance angiography (MRA) revealed multifocal stenoses and extensive collateral formation involving the internal carotid (ICA), middle cerebral (MCA), anterior cerebral (ACA), and posterior cerebral arteries (PCA). The right MCA demonstrated markedly reduced opacification, consistent with high-grade stenosis or occlusion, with additional areas of flow attenuation in multiple vascular territories. These findings raised initial suspicion for a vasculitic process, although the presence of extensive collateralization also suggested a chronic underlying arteriopathy (Figures 5A–C). Initial laboratory investigations were within normal limits except for borderline low albumin, low iron saturation, and mildly elevated inflammatory markers (Supplementary Table 1). The patient denied any history suggestive of seizures, autoimmune disease, or systemic symptoms. Physical and neurological examinations were unremarkable. Notably, a significant family history was present, as her brother had been diagnosed with MMD following an ischemic event and had remained stable on aspirin. She also reported long-standing menorrhagia and was on oral iron supplementation.

A full stroke investigation was initiated, including autoimmune and infectious panel screening (Supplementary Table 2) and lumbar puncture for cerebrospinal fluid (CSF) analysis (Supplementary Table 3). Pending digital subtraction angiography (DSA), aspirin was withheld to minimize procedural bleeding risk.

Although multifocal stenoses and collateral formation initially raised suspicion for CNS vasculitis, several findings supported a non-inflammatory etiology. The patient had no systemic inflammatory symptoms, cerebrospinal fluid analysis was non-inflammatory, and autoimmune and infectious workup were negative. Furthermore, the presence of extensive collateralization suggested chronic vascular remodeling rather than an acute inflammatory process. These features, together with the positive family history, favored a diagnosis of MMD over CNS vasculitis.

Upon multidisciplinary review, the overall clinical, laboratory, and imaging findings made vasculitis less likely, particularly in the absence of systemic inflammatory features, non-inflammatory CSF findings, and evidence of chronic vascular remodeling. MMD, atherosclerosis, and arterial dissection were considered as differential diagnoses.

DSA revealed severe stenosis at the terminus of the right ICA with extensive lenticulostriate collateral formation and only faint distal MCA opacification. A fetal-type posterior communicating artery was present, but the posterior cerebral artery did not supply the anterior circulation retrogradely. On the left, the ICA terminated at the ophthalmic artery with no distal opacification; however, the left MCA and ACA territories were supplied via robust retrograde flow from the PCA. These findings were diagnostic of MMD (Figures 6A and 6B).

The patient started on aspirin 100 mg daily for secondary stroke prevention. Surgical revascularization via superficial temporal artery–to–middle cerebral artery (STA–MCA) bypass was discussed, but the patient and her family chose to defer surgery for outpatient follow-up. She remained admitted for six days and was discharged back neurologically stable with marked improvement in her visual symptoms. At two-month follow-up, she remained asymptomatic and continued multidisciplinary follow-up with neurology, neurosurgery, and genetics teams as part of her long-term management plan.

## 3. DISCUSSION

MMD is a chronic, progressive occlusive cerebrovascular disorder that primarily affects the intracranial internal carotid arteries and their proximal branches. The disease is characterized by the compensatory development of fragile collateral vessels, producing the hallmark "puff of smoke" appearance on cerebral angiography.^8–10oka^ Although MMD is most prevalent in East Asian populations, particularly Japan and Korea, it is increasingly recognized worldwide.^4,11^

In this report, we described a 38-year-old woman with no prior medical history but a positive family history of MMD. She presented with bilateral visual disturbances and headaches, symptoms attributable to occipitoparietal ischemia confirmed on neuroimaging. While ischemic strokes and transient ischemic attacks (TIAs) represent the most common presentations of MMD in adults, atypical symptoms such as visual changes have also been documented in other case reports,^8,12^ highlighting the potential for diagnostic delay.

HR-VWI in our case demonstrated circumferential enhancement, which initially raised concern for CNS vasculitis. However, vessel wall enhancement patterns in MMD are variable and may include concentric or patchy enhancement, reflecting chronic vascular remodeling. Importantly, these findings are not specific and may overlap with those observed in inflammatory vasculopathies. Therefore, HR-VWI findings should not be interpreted in isolation but rather integrated with clinical, laboratory, and angiographic data to reach an accurate diagnosis.^6,13^ Similar diagnostic dilemmas have been reported in the literature, underscoring the need for multimodal assessment.^14^ In our patient, bilateral ICA stenosis with robust lenticulostriate and fetal posterior communicating artery (PCom) collateralization favored a diagnosis of MMD.

The presence of a positive family history further supported the diagnosis, as familial forms of MMD have been well described and are often associated with genetic susceptibility. Mutations in the RNF213 gene have been strongly linked to familial and sporadic cases, particularly in East Asian populations, although their role in other populations is increasingly recognized.^15–17^ While genetic testing was not performed in this case, it may be considered in patients with a strong family history to support diagnosis and guide counseling.

Management of MMD differs fundamentally from that of CNS vasculitis. Immunosuppressive therapy, which is the mainstay of treatment for vasculitis, is ineffective in MMD and may lead to unnecessary delays in appropriate management. Antiplatelet therapy is commonly used for secondary stroke prevention in patients with ischemic presentation, although supporting evidence remains largely observational. Surgical revascularization, including direct or indirect bypass procedures, is an important treatment option in selected patients, particularly those with recurrent ischemia or impaired cerebral perfusion.^8,16^ Similar to other reports, our patient was initiated on aspirin and referred for neurosurgical evaluation, with surgical revascularization under consideration.^18,19^ Treatment decisions should be individualized based on clinical presentation, hemodynamic status, and surgical suitability.^20,21^ In our case, aspirin was initiated for secondary prevention, and surgical revascularization was discussed but deferred based on patient preference and clinical stability.

This case highlights the importance of distinguishing non-inflammatory arteriopathies from true inflammatory vasculitis. In our patient, the absence of systemic inflammatory features, non-inflammatory CSF findings, negative autoimmune workup, and evidence of chronic vascular remodeling were key factors supporting the diagnosis of MMD. These findings emphasize the importance of a structured, multimodal diagnostic approach.

This case emphasizes the importance of maintaining a broad differential diagnosis when evaluating young patients with stroke-like symptoms. Early recognition is essential to avoid misdiagnosis, prevent unnecessary immunosuppressive therapy, and enable timely referral for appropriate management.

## 4. CONCLUSION

MMD can closely mimic CNS vasculitis, making accurate diagnosis essential to avoid unnecessary or potentially harmful immunosuppressive therapy. This case illustrates the value of multidisciplinary collaboration in diagnostically complex scenarios. Our patient was managed medically with aspirin, declined surgical revascularization, and remained clinically stable at follow-up without recurrence of neurological symptoms. Clinicians should remain cautious when interpreting vessel wall enhancement in isolation and integrate clinical, imaging, and family history data to guide management.

## ACKNOWLEDGEMENT

The authors have no acknowledgements to declare.

## AUTHORS’ CONTRIBUTIONS

AS and HE contributed to drafting the Introduction. AS and AHA contributed to drafting the Discussion. VS and AE contributed to drafting the Case Presentation. EA contributed to the Case Presentation and was responsible for the selection and interpretation of the radiological images. AH and GA provided supervision. All authors reviewed and approved the final manuscript.

## CONFLICT OF INTEREST

The authors declare that they have no conflicts of interest.

## DISCLOSURE OF AI USE

No artificial intelligence tools were used in the preparation of this manuscript.

## DATA AVAILABILITY

The data supporting the findings of this case report are contained within the article and its supplementary materials. Additional patient-level data are not publicly available to protect patient confidentiality.

## FUNDING

The authors received no specific funding for this work.

## ETHICAL APPROVAL

This case was managed at Hamad General Hospital, Doha, Qatar, a tertiary public-sector healthcare facility. Institutional approval for publication was obtained through the Medical Research Center, Hamad Medical Corporation (MRC approval number: MRC-04-25-928). Written informed consent for participation and publication of associated clinical details and imaging was obtained from the patient.

## SUPPLEMENTARY MATERIALS

**Supplementary Table 1. Summary of the patient’s routine blood chemistry results at the time of initial presentation in the emergency department.**

**Supplementary Table 2. Summary of the patient’s autoimmune and infectious panel results.**

**Supplementary Table 3. Summary of the patient’s cerebrospinal fluid (CSF) analysis at the time of admission.**



## Figures and Tables

**Figure 1.**
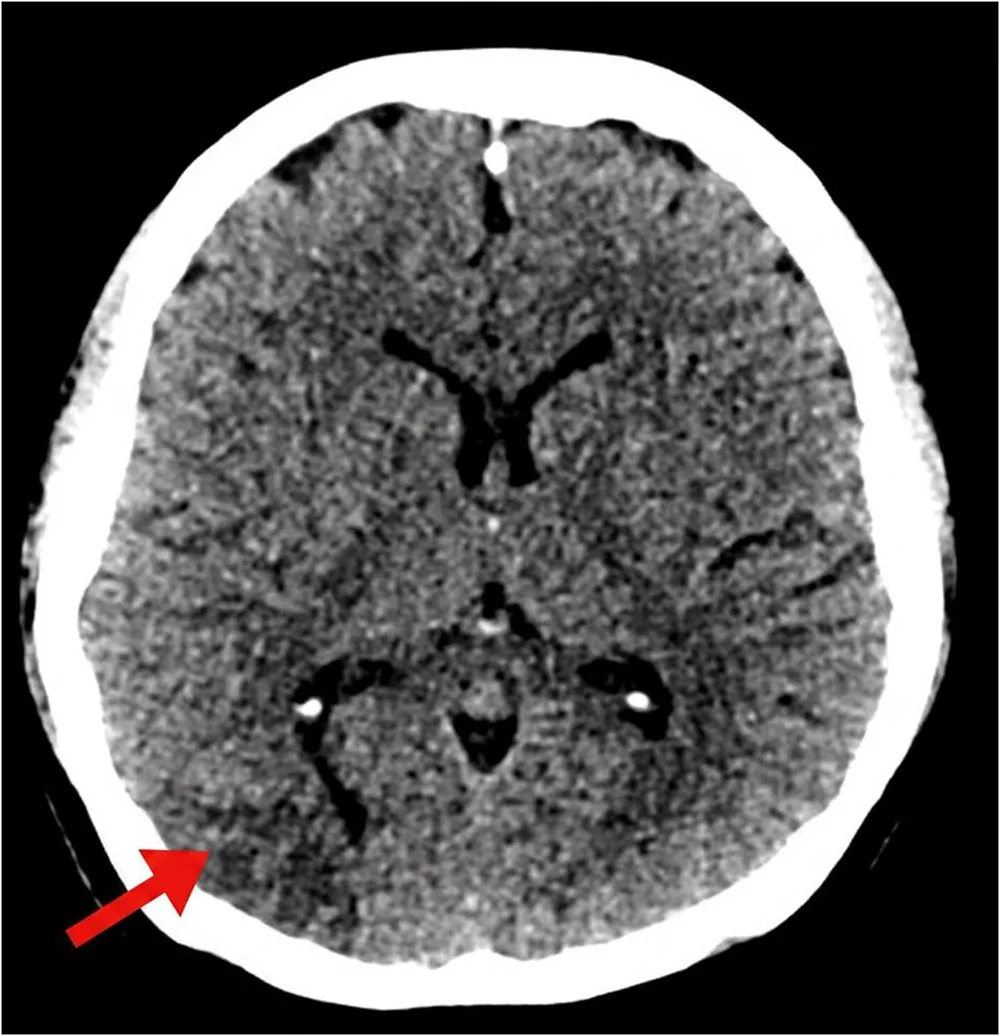
Non-contrast computed tomography (CT) of the brain demonstrating a hypodense lesion in the right occipitoparietal region with loss of gray–white matter differentiation and mild mass effect (red arrow), consistent with subacute infarction.

**Figure 2.**
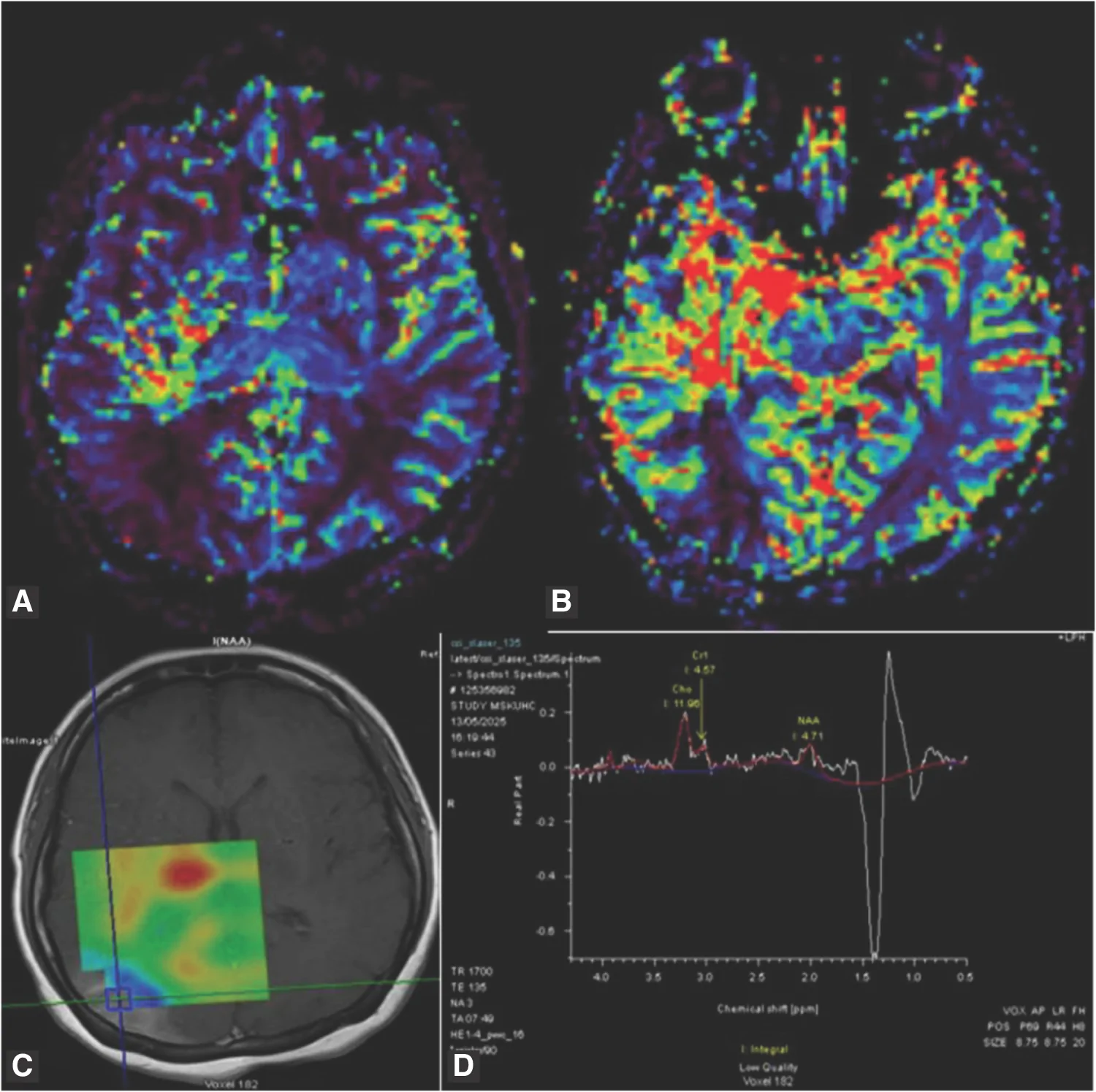
Magnetic resonance perfusion imaging and spectroscopy: (A) Cerebral blood flow (CBF) map demonstrating reduced perfusion in the right occipitoparietal region (arrow). (B) Cerebral blood volume (CBV) map showing reduced blood volume (arrow). (C) Perfusion map with region of interest placement. (D) Magnetic resonance spectroscopy demonstrating reduced N-acetylaspartate (NAA) peak, consistent with neuronal loss.

**Figure 3.**
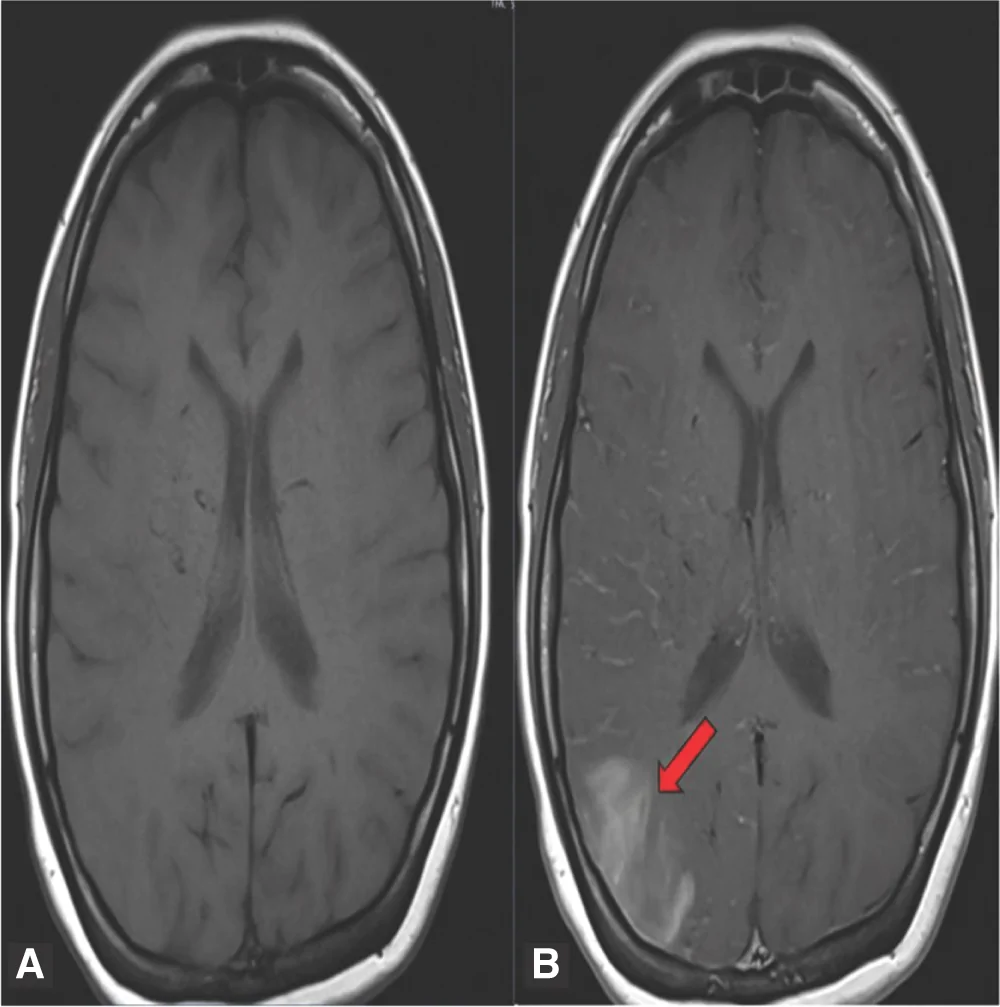
Magnetic resonance imaging (MRI) of the brain: (A) T1-weighted image showing isointense signal in the right occipitoparietal region. (B) Post-contrast T1-weighted image demonstrating cortical and subcortical enhancement, consistent with subacute infarction (red arrow).

**Figure 4.**
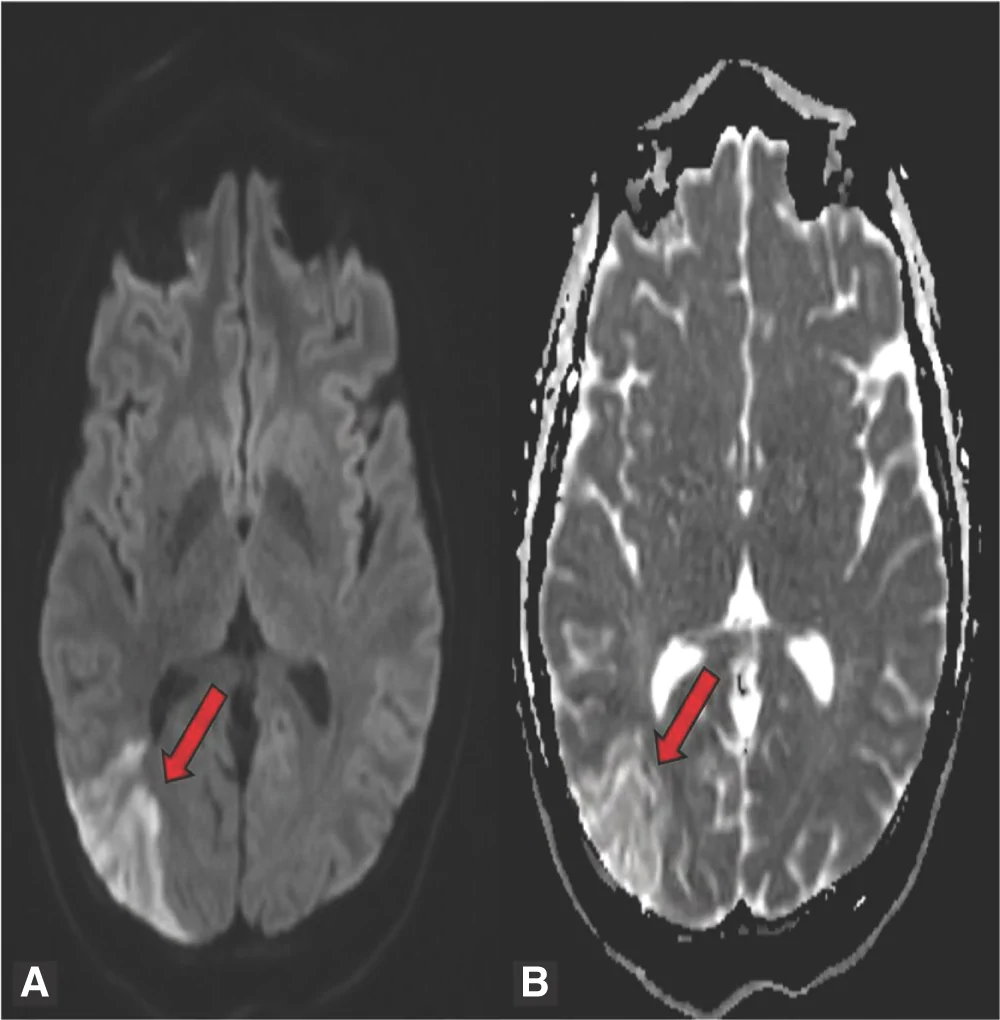
MRI of the brain: (A) diffusion-weighted imaging (DWI) and (B) apparent diffusion coefficient (ADC) map showing restricted diffusion in the right occipitoparietal region, consistent with acute or subacute infarction (red arrows).

**Figure 5.**
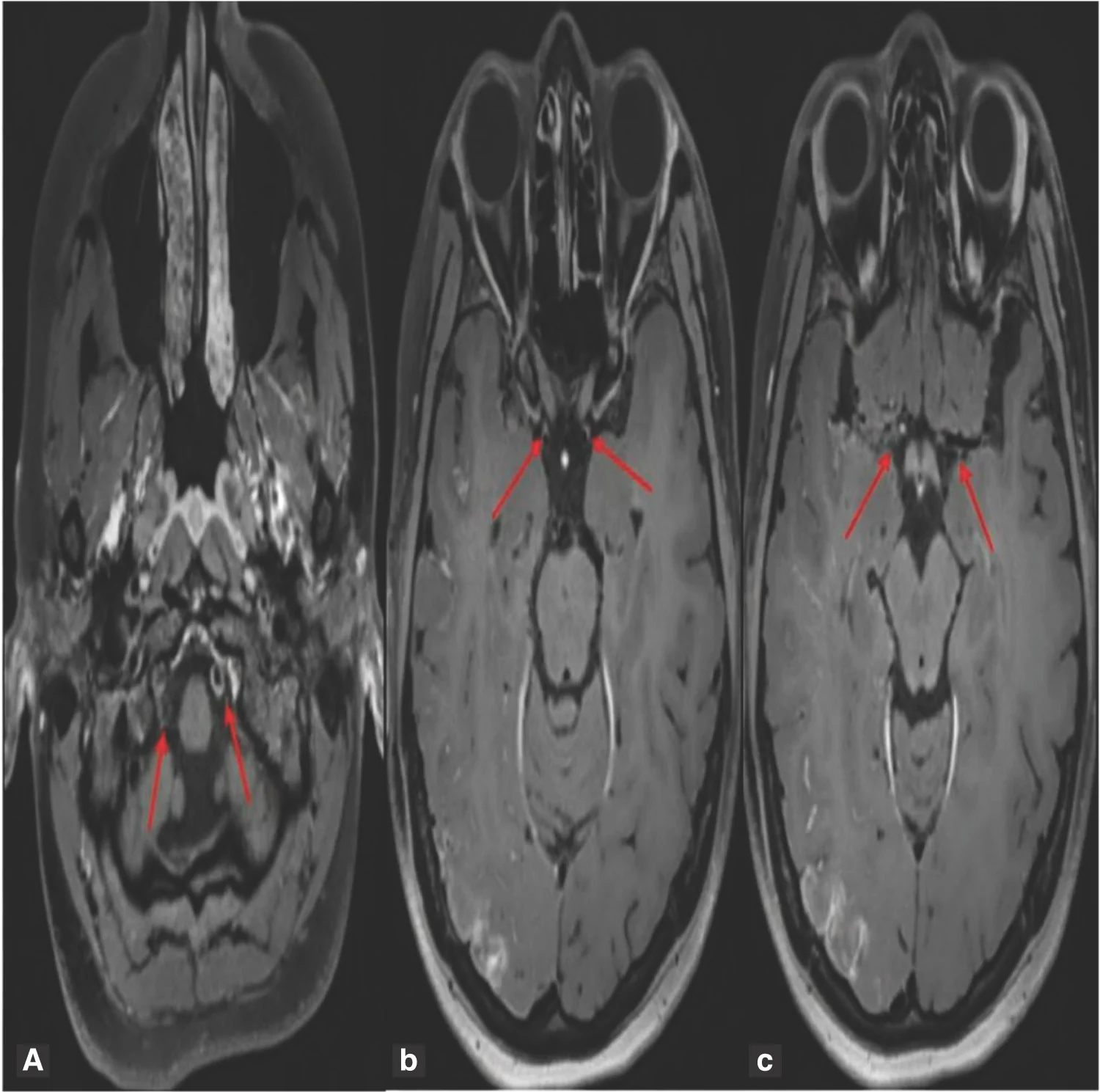
High-resolution vessel wall MRI, demonstrating (A) circumferential enhancement of intracranial arteries, including the vertebral arteries, (B) internal carotid arteries (ICAs), and (C) middle cerebral arteries (MCAs) (red arrows). These findings are non-specific and may be seen in both inflammatory vasculopathies and non-inflammatory arteriopathies, such as Moyamoya disease; therefore, interpretation requires correlation with clinical, laboratory, and angiographic findings.

**Figure 6.**
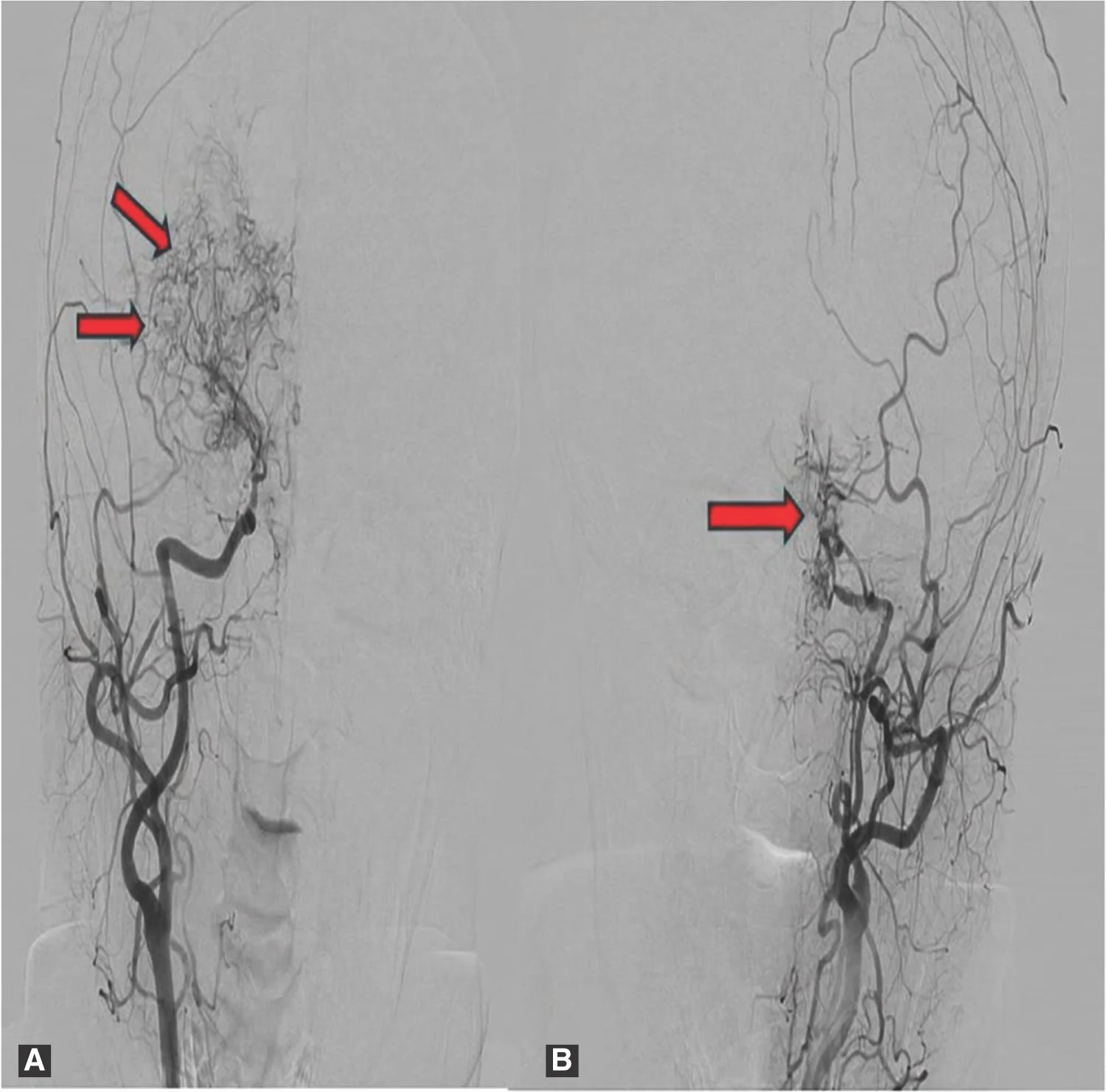
Digital subtraction angiography (DSA): (A) right internal carotid artery (ICA) showing stenosis at its terminus with lenticulostriate collaterals, and (B) left ICA terminating at the ophthalmic artery, with middle cerebral artery (MCA) and anterior cerebral artery (ACA) territories supplied retrogradely from the posterior cerebral artery (PCA) (red arrows).

**Table 1.** Summary of key diagnostic findings. Detailed laboratory, autoimmune/infectious, and cerebrospinal fluid results are provided in Supplementary Tables 1–3.

Category	Findings
Cerebrospinal fluid (CSF)	Non-inflammatory (normal cell count, normal protein, no oligoclonal bands)
Autoimmune panel	Negative (ANA, ANCA, anti-dsDNA, antiphospholipid antibodies)
Infectious workup	Negative (viral PCR panel, bacterial cultures, TB screening)
Inflammatory markers	Mildly elevated CRP and ESR
Metabolic profile	Mild hypoalbuminemia and low iron saturation; otherwise, unremarkable
Family history	Positive for Moyamoya disease in a first-degree relative
