# Supplementary material for: Moyamoya disease masquerading as CNS vasculitis: Diagnostic challenges and imaging insights
Source: Qatar Med J. 2026 Sep 22;2026(3):68. doi: 10.5339/qmj.2026.68 (PMC13622694; doi:10.5339/qmj.2026.68)
Supplement: Supplementary file 1 [file qmj-2026-03-068.supp1.docx]

Supplementary Materials

Supplementary Table 1. Summary of the patient’s routine blood chemistry results at the time of initial presentation in the emergency department.

| Parameter | Result | Reference range | Units |
| --- | --- | --- | --- |
| Urea | 2.30 | 2.5–7.80 | mmol/L |
| Creatinine | 50 | 44–80 | µmol/L |
| Sodium | 140 | 135.0–145.0 | mmol/L |
| Potassium | 3.9 | 3.50–5.30 | mmol/L |
| Chloride | 105 | 96.0–110.0 | mmol/L |
| Bicarbonate | 25 | 22.2–29.0 | mmol/L |
| Calcium (Total) | 2.24 | 2.10–2.55 | mmol/L |
| Corrected calcium | 2.32 | 2.10–2.55 | mmol/L |
| Bilirubin (Total) | 9 | 3.40–20.50 | µmol/L |
| Total protein | 69 | 64.0–83.0 | g/L |
| Albumin | 30 | 35.0–50.0 | g/L |
| Alkaline phosphatase (ALP) | 103 | 40.0–150.0 | U/L |
| Alanine aminotransferase (ALT) | 11 | 0.0–55.0 | U/L |
| Aspartate aminotransferase (AST) | 12 | 5.0–34.0 | U/L |
| Iron | 10 | 6.0–35.0 | µmol/L |
| Total iron binding capacity (TIBC) | 88 | 45–80 | % |
| Transferrin | 3.5 | 2.0–3.6 | g/L |
| Ferritin saturation (Fe Sat) | 11 | 15.0–45.0 | % |
| Glucose | 5.7 | 3.3–5.5 | mmol/L |
| C-reactive protein (CRP) | 6.8 | 0.0–5.0 | mg/L |
| Erythrocyte sedimentation rate (ESR) | 25 | 2–37 | mm/hr |
| Total cholesterol | 5.1 | 5.2–5.6 | mmol/L |
| Triglyceride | 1.2 | >1.7 | mmol/L |
| High-density lipoprotein (HDL) | 1.2 | <1.0 | mmol/L |
| Low-density lipoprotein (LDL) | 3.4 | >2.59 | mmol/L |
| Immunoglobulin G (IgG) | 10.16 | 6.10–16.16 | g/L |

**Supplementary Table 2. Summary of the patient’s autoimmune and infectious panel results.**

| Test | Result | Reference range / Interpretation | Units |
| --- | --- | --- | --- |
| Anti-dsDNA antibody | 1.00 | Negative | IU/mL |
| Anti-cardiolipin IgG | <0.50 | Negative | GPL |
| Anti-cardiolipin IgM | 8.50 | Negative | MPL |
| Antinuclear cytoplasmic antibody (ANCA) | Negative | Negative | — |
| Anti-myeloperoxidase (MPO) Ab | <0.20 | Negative | IU/mL |
| Anti-proteinase 3 antibody | <0.20 | Negative | IU/mL |
| Anti-beta 2 glycoprotein IgG | 0.60 | Negative | U/mL |
| Anti-beta 2 glycoprotein IgM | <2.90 | Negative | U/mL |
| ANA–connective tissue disease (CTD) panel | Negative | Negative | — |
| C3 complement | 1.63 | Normal | g/L |
| C4 complement | 0.32 | Normal | g/L |
| Quantiferon TB gold plus | Negative | Negative | — |
| Hepatitis B surface antigen | Non-reactive | Non-reactive | — |
| Hepatitis C antibody | Non-reactive | Non-reactive | — |
| HIV Ag/Ab combo | Non-reactive | Non-reactive | — |
| Fungal culture | Negative | Negative | — |
| Adenovirus | Negative | Negative | — |
| Enterovirus | Negative | Negative | — |
| Cytomegalovirus (CMV) | Negative | Negative | — |
| Parechovirus | Negative | Negative | — |
| Human herpes virus type 6 (HHV-6) | Negative | Negative | — |
| Herpes simplex virus type 1 (HSV-1) | Negative | Negative | — |
| Herpes simplex virus type 2 (HSV-2) | Negative | Negative | — |
| Varicella zoster virus (VZV) | Negative | Negative | — |
| Escherichia coli K1 | Negative | Negative | — |
| Haemophilus influenzae | Negative | Negative | — |
| Listeria monocytogenes | Negative | Negative | — |
| Neisseria meningitidis (encapsulated) | Negative | Negative | — |
| Streptococcus agalactiae | Negative | Negative | — |
| Streptococcus pneumoniae | Negative | Negative | — |
| Cryptococcus neoformans/gattii | Negative | Negative | — |

**Supplementary Table 3. Summary of the patient’s cerebrospinal fluid (CSF) analysis at the time of admission.**

| Parameter | Result | Units |
| --- | --- | --- |
| Volume | 0.5 | mL |
| Color | Colorless | — |
| Appearance | Clear | — |
| Red blood cells (RBC) | 5 | /µL |
| Total nucleated cells | 1 | /µL |
| CSF glucose | 4.12 | mmol/L |
| CSF protein | 0.40 | g/L |
| CSF protein (alternative measure) | 287 | mg/L |
| CSF immunoglobulin G (IgG) | 38 | mg/L |
| CSF IgG oligoclonal bands | Not detected | — |
| CSF IgG index | 0.5 | — |
| CSF culture | Negative | — |
| CSF cryptococcal culture | Negative | — |
